# Evidence-based recommendations for the management of anterior cruciate ligament (ACL) rupture

**DOI:** 10.1016/j.berh.2019.01.018

**Published:** 2019-02

**Authors:** Stephanie R. Filbay, Hege Grindem

**Affiliations:** a Arthritis Research UK Centre for Sport, Exercise and Osteoarthritis, Nuffield Department of Orthopaedics, Rheumatology & Musculoskeletal Sciences, Botnar Research Centre, University of Oxford, Oxford, OX3 7LD, UK; b Oslo Sport Trauma Research Center, Department of Sports Medicine, Norwegian School of Sport Sciences, PB 4014 Ullevål Stadion, Oslo, 0806, Norway

**Keywords:** Anterior cruciate ligament reconstruction, Clinical recommendations, Evidence-based practice, Knee osteoarthritis, Patient-centered care, Quality of life, Rehabilitation, Return to sport

## Abstract

Anterior cruciate ligament (ACL) rupture occurs most commonly in young and active individuals and can have negative long-term physical and psychological impacts. The diagnosis is made with a combination of patient's history, clinical examination, and, if appropriate, magnetic resonance imaging. The objectives of management are to restore knee function, address psychological barriers to activity participation, prevent further injury and osteoarthritis, and optimize long-term quality of life. The three main treatment options for ACL rupture are (1) rehabilitation as first-line treatment (followed by ACL reconstruction (ACLR) in patients, who develop functional instability), (2) ACLR and post-operative rehabilitation as the first-line treatment, and (3) pre-operative rehabilitation followed by ACLR and post-operative rehabilitation. We provide practical recommendations for informing and discussing management options with patients, and describe patient-related factors associated with a worse ACL-rupture outcome. Finally, we define evidence-based rehabilitation and present phase-specific rehabilitation recommendations and criteria to inform return to sport decisions.

## Introduction

In the USA alone, 250,000 individuals suffer an anterior cruciate ligament (ACL) rupture per year [1]. .These injuries commonly incur during sports and exercise, and clinical practice patterns for ACL rupture management differ across the globe. In North America, athletes with an ACL rupture most often undergo surgical ACL reconstruction (ACLR). However, this is not the standard of management in all countries. Regardless of surgical intervention or not, there has been an increased focus on the importance of performing evidence-based rehabilitation. In this context, the term evidence-based rehabilitation refers to exercise therapy, which may be augmented with other modalities that have scientific evidence of benefit. Exercise therapy includes components, such as resistance training, neuromuscular exercise, high-level dynamic functional tasks and sport-specific training. This chapter presents the best-evidence recommendations for the clinician reader, who treats patients with ACL rupture. We provide a guide to injury diagnosis and management, with a focus on rehabilitation and return to sport decisions. This chapter also includes a best-evidence summary of the consequences of ACL rupture, outcomes with different treatment strategies, and patient-related factors that are associated with outcome. This information will provide clinicians with the necessary knowledge to inform and make shared management decisions with their patients, with the overarching aim of optimizing function and quality of life (QOL).

### How do you diagnose an ACL rupture?

To accurately diagnose an ACL rupture, the clinician will combine information from a patient history, clinical examination and, if appropriate, imaging. ACL ruptures often occur with concomitant injury to menisci, cartilage, or other knee ligaments. Special consideration should be taken to diagnose substantial concomitant injuries accurately.

#### History

An ACL rupture should always be suspected if the patient reports (1) an injury mechanism that involves deceleration/acceleration in combination with a knee valgus load, (2) hearing or feeling a “pop” at the time of injury, or (3) hemarthrosis within 2 h of injury [2].

#### Clinical examination

Several clinical tests can be used to detect an ACL rupture. The Lachman test is the most accurate clinical diagnostic test, with a pooled reported sensitivity of 85% and specificity of 94% [3]. The anterior drawer test has high sensitivity and specificity for chronic ACL ruptures (92% sensitivity and 91% specificity), but lower accuracy for acute cases [3]. When positive, the pivot shift test is a very clear indication of an ACL rupture (98% specificity). A negative test is, however, not sufficient to rule the injury out (24% sensitivity) [3].

#### Magnetic resonance imaging (MRI)

In experienced assessors, the combination of patient history and clinical examination will often be sufficient to diagnose an ACL rupture. However, pain and effusion in an acute setting may make it difficult to detect the injury during clinical examination. Misdiagnosis is common; half of patients with an acute ACL rupture were misdiagnosed as an uncomplicated knee sprain in an orthopedic emergency unit [4]. Repeated clinical examination or MRI in the subacute phase may therefore be necessary to rule the injury out. The diagnostic accuracy of MRI is comparable to that of the Lachman test [5]. For patients with suspected ACL rupture, MRI holds value as (1) an adjunct when the clinical diagnosis is uncertain; and (2) part of the assessment of concomitant knee injuries that may be harder to diagnose clinically (e.g., meniscal and cartilage injuries).

#### Differential diagnosis and concomitant injuries

Following substantial acute knee trauma, the Ottawa Knee Rule should be used to rule out fracture [6]. The rule is designed to accurately exclude knee fractures (sensitivity 98.5%), but is not sufficient to rule a fracture in (specificity 48.6%) [6]. Patients who have at least one positive answer on any of five questions (Table 1) should be considered for x-ray imaging. Adolescents who present with acute effusion after rotational knee trauma should also be carefully assessed to rule out patellar dislocation [4], [7].

**Table 1:** Questions in the Ottawa knee rule [8].

Is the patient 55 years or older?
Is there isolated tenderness of the patella?
Is there tenderness of the head of the fibula?
Is the patient unable to flex the knee to 90°?
Is the patient unable to bear weight for four steps?

ACL ruptures often occur with concomitant ligament sprains, meniscus tears, bone marrow lesions, articular cartilage injuries, and intra-articular fractures [4]. The rates of concomitant lateral collateral ligament (LCL) and posterior cruciate ligament (PCL) injuries are generally low, while concomitant medial collateral ligament (MCL) injuries and meniscal tears are common (prevalence of 30% and 42%, respectively) [4].

### What are the consequences of an ACL rupture?

Once the diagnosis is clear, the clinician should inform the patient of the injury and known consequences. For many individuals, their ACL-injured knee will never feel as it did before the injury. More than five years after ACL rupture, knee pain, symptoms, recreational limitations, and impaired QOL are common [9], [10]. Additionally, an alarming number of individuals with ACL rupture will develop symptomatic knee osteoarthritis during young- or middle-adulthood [11]. Many individuals do not return to sport and adopt a physically inactive lifestyle, and fear of re-injury is likely to be a contributing factor in this decision [12], [13], [14]. Of further concern is the high rate of re-injury, which is associated with worse long-term outcome [15], [16], [17]. Collectively, these factors can have a detrimental impact on the QOL of individuals who were highly active before sustaining an ACL rupture and often rely on their ability to be active for a fulfilling and satisfying QOL [18], [19]. However, not all individuals have poor outcomes after ACL rupture. This highlights the importance of identifying modifiable risk factors for poor outcome in ACL-injured individuals, and implementing personalized management strategies to optimize long-term outcome and QOL across the lifespan.

### What are the objectives of ACL rupture management?

The objectives of management for an individual with ACL rupture are to (1) restore knee function, (2) address psychological barriers to resuming activity participation, (3) prevent further knee injury and reduce the risk of knee osteoarthritis, and (4) optimize long-term QOL.

#### Restore knee function

Treatments to achieve this objective are targeted toward the specific impairments of the patient. Establishing realistic and specific goals, and accurate assessment of current impairments that need to be addressed to reach these goals, are therefore key. Typical impairments after injury include varying degrees of muscle strength deficits, altered movement patterns, decreased knee joint proprioception, and increased passive knee laxity. Collectively, these impairments contribute to the varying degrees of functional knee instability that patients with ACL rupture experience.

#### Address psychological barriers to resuming activity participation

Although the functional status of the knee is associated with whether the patient returns to sport [20], problems with the injured knee is only the third most frequently cited reason for not returning to sport [13]. More patients attribute not returning to sport to a fear of re-injury or a lack of trust in the knee. Continued follow-up and support throughout the return to sport period may therefore be needed to ease the transition back to sports.

#### Prevent further knee injury and reduce the risk of knee osteoarthritis

The main threats to future knee health are knee re-injury and the development of posttraumatic knee osteoarthritis. To date, no randomized trials exist to support interventions that can eliminate these risks. The risk for knee re-injury is lower in those who (1) do not participate in sports with frequent pivoting and cutting, (2) complete rehabilitation to the point where they pass functional return to sport criteria before returning to pivoting sports, and (3) return to pivoting sports later than 9 months after an ACLR [21], [22]. Additionally, neuromuscular training programs are highly effective in reducing the risk of knee injury in a primary setting [23]. To reduce the risk for knee re-injury, the clinician should provide patient-education that includes information on the probable benefit of activity modification. In patients who make an informed decision to pursue participation in pivoting sports following ACLR, the treatment strategy should include at least 9 months postoperative rehabilitation, return to sport only after passing specific criteria, and continued performance of neuromuscular training programs after return to sport.

The risk for knee osteoarthritis is higher in those with higher BMI, those who are physically inactive, and those with quadriceps muscle weakness [24], [25]. Although physical inactivity and obesity are not common concerns in the young and active people who usually sustain ACL ruptures, sustaining a knee injury in youth increases the risk of becoming physically inactive and gaining fat mass in the longer term [26]. These modifiable risk factors should therefore be targeted after ACL rupture. Regaining quadriceps muscle strength is an essential goal of rehabilitation after ACL rupture and can be achieved with heavy resistance strength training [27]. Because many athletes do not return to their preinjury level of sport, guidance on alternative modes of physical activity may be a key factor to prevent physical inactivity. Finally, patients who already have a high BMI at the time of ACL rupture should receive support to adopt healthy weight-loss strategies.

#### Optimize long-term quality of life

Maintaining participation in a satisfying form of physical activity is a key determinant of long-term QOL following ACLR [19]. Negotiating fear of re-injury and decisions surrounding continuation or cessation of sport participation are also closely related to long-term QOL after ACLR [19]. Individuals who return to preinjury sport after ACL rupture report better health-related and knee-related QOL 5–20 years later, compared with those who do not return to sport [18]. Physical activity and sport participation play an important role in the lives of individuals with ACL rupture, and the inability to be physically active has a detrimental effect on the QOL of those who develop symptomatic post-traumatic knee osteoarthritis [28]. A key objective is to reduce the negative impact the ACL-ruptured knee has on a person's current and future QOL. This will require a personalized approach in line with the individual's goals, values, and life priorities. The clinician should be aware that goals, values, and life priorities are likely to change over time, and an individual with ACL rupture may benefit from guidance and support to adapt to changing life circumstances and manage their knee throughout various stages of their life [19].

### What are the common management strategies for ACL rupture?

The treatment for patients with ACL rupture needs to be individualized and several options are currently in use in clinical practice. In this chapter, we present three main options for the reader (Table 2). Considering methods for rehabilitation and surgical techniques can vary, rehabilitation clinicians and surgeons should both be a part of the discussion of best treatment for the individual patient. Most of the scientific literature in his area is centered on the debate of whether patients who undergo ACLR have better outcomes than those who are treated without ACLR. In the following section, we present the current evidence on this topic.

**Table 2:** Main management options for treatment of ACL rupture.

(1) Rehabilitation as the first-line treatment (followed by ACLR and postoperative rehabilitation if the patient develops functional instability).
(2) ACLR as the first-line treatment, followed by postoperative rehabilitation.
(3) Preoperative rehabilitation followed by ACLR and postoperative rehabilitation.

### Do the outcomes of ACL rupture differ depending on management strategy?

There are a number of literature reviews comparing outcomes between individuals that are ACL-deficient or have had an ACLR [9], [29], [30], [31], [32]. It is important to note that most studies included in these reviews do not reflect best practice for nonoperative management of ACL rupture. For example, many of the nonoperatively managed patients in these studies received a diagnostic knee arthroscopy, some were advised to reduce activity levels, and rehabilitation was often not monitored, of a low intensity or short duration, or included post-injury immobilization with a brace or cast [9], [29], [30], [31], [32], [33]. Despite this, these literature reviews report similar outcomes in ACL-deficient and ACL-reconstructed groups, including similar patient-reported outcomes, knee function, activity levels, QOL [9], [29], [30], [33] and either no difference in radiographic osteoarthritis prevalence [29] or a slightly increased prevalence following ACLR [30], [32].

There is a notable shortage of high quality research comparing outcomes following the management of patients with an ACL rupture with high-quality rehabilitation compared to ACLR. A recent review of all randomized controlled trials (RCTs) for ACL injury identified only 1 (The KANON Trial) out of 412 trials that compared outcomes following ACL management with rehabilitation plus optional delayed ACLR vs. ACLR and postoperative rehabilitation [34]. The two groups had similar two-year and five-year self-reported physical activity levels, rates of meniscus surgery, symptoms, pain, QOL, and radiographic joint changes [35], [36]. Notably, there was also no difference in outcomes between patients, who had an early ACLR, patients managed with rehabilitation alone, and those initially managed with rehabilitation who underwent a delayed ACLR (51% after five years) [35], [36].

Several relevant recent studies did not feature in the previous reviews. A recent cohort study compared outcomes between patients with an ACL rupture managed with strength and neuromuscular training or ACLR followed by postoperative rehabilitation. The treatment groups had similar five-year functional and radiographic outcomes, but the ACLR group had more joint effusion, better self-reported knee function, and lower self-reported fear [37]. Furthermore, Kovalak et al. (2018) found no group difference in time to return to regular physical activity, eight-year knee function, strength or QOL between athletes who were managed with neuromuscular training alone or with ACLR [38]. Van Yperen et al. (2018) compared 20 year outcomes in 50 high-level athletes with an ACL rupture managed with structured rehabilitation and lifestyle modifications or ACLR (due to persistent instability after 3-months of nonoperative management). Despite the fact that the ACLR group had less knee laxity, there were no between-group differences in meniscectomy rates, radiographic osteoarthritis, and functional outcomes [39]. Combined, these recent studies support a larger body of evidence demonstrating that on average, individuals experience similar functional, radiographic, and patient-reported outcomes after ACL rupture, irrespective of management with rehabilitation alone or with ACLR.

#### Knee laxity and functional instability

Knee *laxity* refers to the passive intra-articular translation available in the tibiofemoral joint (i.e., assessed manually or by a device) whereas *functional instability* refers to the patient's perception of an “unstable” knee [40]. The magnitude of knee laxity is of questionable clinical relevance given that it does not account for dynamic joint control afforded by the surrounding musculature. Studies that report more knee laxity in ACL-deficient individuals (compared to ACL-reconstructed individuals), also find similar self-reported knee instability, functional outcomes and physical activity levels between groups [30], [39]. Research suggests that despite greater laxity in ACL-deficient knees compared to ACLR knees, *functional stability* can be achieved through neuromuscular training [31]. Despite common misconceptions to the contrary, research suggests that it is possible for an individual who suffers an ACL rupture to return to sport following management with rehabilitation alone [30], [36], [41]. Several studies have found no difference in physical activity levels or return to sport rates between patients managed with ACLR and those managed with rehabilitation [30], [36], [41].

#### Additional knee injury and surgery

Meniscus or cartilage damage at the time of ACLR is associated with worse outcomes and higher rates of osteoarthritis [11], [42], [43], [44], [45], [46], [47], [48], [49]. Reducing the risk for subsequent meniscus and cartilage injury should therefore be a key priority when managing individuals with an ACL rupture.

Subsequent knee injury is common following ACLR; one third of young individuals who undergo ACLR experience a second ACL tear [15] and 27% experience a third within 2–9 years after revision ACLR [50]. This is concerning, considering a second ACL injury is associated with meniscus and/or cartilage damage in approximately 90% of individuals [51] and ACLR revision is associated with more pain and symptoms, reduced function, inactivity, higher rates of osteoarthritis, and worse QOL compared to primary ACLR [16], [17], [52].

A common belief is that early ACLR prevents additional meniscus and cartilage injury. However, studies referenced to support this belief are typically retrospective reviews of surgical records, and show more severe joint injury in patients presenting for ACLR months or years after ACL rupture, following unknown/no rehabilitation [53]. On the other hand, evidence from prospective studies do not suggest management with rehabilitation as the first-line treatment results in more joint injury compared with management with early ACLR [53]. Grindem et al. (2014) reported that knee re-injury was more common in patients who chose ACLR (24%) compared to those who chose rehabilitation alone (9%). This difference was not significant after accounting for group differences, suggesting that being young and active has a larger impact on re-injury risk than the patient's chosen treatment [54]. Additionally, the KANON Trial found a similar proportion of patients underwent meniscus surgery between ACL treatment groups over a five-year follow-up period [36]. In summary, best available evidence *does not* indicate that an individual is at greater risk of subsequent injury if they are managed with rehabilitation as the first-line treatment as opposed to ACLR.

### What patient-related factors are associated with ACL rupture outcome?

Understanding patient-related factors that are associated with outcomes after ACL rupture can enable the clinician to better tailor the information and management strategy to the individual patient. In those who are treated with ACLR, younger athletes, men, and elite athletes are more likely to return to sport [13]. Athletes under the age of 25 also have higher rates of second ACL ruptures than older athletes [55]. Compared to those who have concomitant meniscal or cartilage pathology, patients with isolated ACL ruptures are likely to be more physically active, have better knee function, and less pain 2–6 years after ACLR [56], [57], [58].

Patients who are better prepared, both psychologically and functionally, prior to ACLR also have better outcomes after an ACLR. Preoperative factors associated with better postoperative outcomes include optimism, self-efficacy, greater quadriceps muscle strength, and passive knee extension range of motion [59], [60], [61]. These findings highlight the importance of preoperative rehabilitation, which has been associated with better postoperative patient-reported function and activity level compared to no or limited rehabilitation prior to ACLR [62], [63], [64].

Fewer studies are available on factors that are associated with outcomes following rehabilitation alone, and it is largely unknown how and if these factors interact with treatment choice. An exploratory analysis of the KANON trial showed that undergoing repeat knee surgery was associated with worse five-year pain, symptoms, function in sport, and QOL, and regardless of treatment [57]. However, meniscal injury, chondral damage and worse pain, symptoms, function in sport and QOL at baseline was only associated with worse five-year outcomes (symptoms, function in sport, and QOL) in the group who had early ACLR and rehabilitation [57].

Recently, Grindem et al. [65] proposed predictive models for successful 2-year outcome in nonprofessional pivoting sport athletes, who were treated for ACL rupture with rehabilitation as the first-line treatment. An athlete was classified as having a successful outcome if he or she avoided functional instability leading to late ACLR *and* had self-reported knee function within the normative range of individuals with no history of knee injury. Athletes who were older, women, and individuals with better knee function early after ACL rupture were more likely to have successful two-year outcomes [65]. This information may be used to inform patients of the likelihood of a successful outcome if deciding to pursue management of ACL rupture with rehabilitation alone.

### How should you discuss management options and expectations with your patient?

ACL ruptures can have serious long-term consequences, optimal management requires substantial investment from the patient, and the patient's short- and long-term priorities may be conflicting (i.e., short-term return to sport might be prioritized over long-term knee health). The treatment choice should therefore be a shared decision between the individual patient and the treating health-care professionals. To ensure that the patient makes an informed commitment to a treatment plan, the first step of this process is to provide high-quality information about the injury, short- and long-term consequences, what the different treatment options are, and the patient's likely prognosis with the different treatment options.

#### Expectations

Patients tend to have very high expectations prior to ACLR, which do not match average outcomes. Of 181 patients who awaited ACLR, all patients expected to have almost normal or normal knee function within 12 months of surgery, 91% expected to return to sport within one year of surgery and 98% expected no or only a slight increased risk of knee osteoarthritis after ACLR [66]. These expectations are not realistic; long-term knee symptoms are common [67], only 42% of nonprofessional athletes return to competitive sport following ACLR [13] and as many as 50% develop radiographic knee osteoarthritis within 10 years of ACLR [11]. Although a rigorous informed consent process takes time in a busy clinical schedule, clinicians have a fiduciary duty to inform the patient of the expected outcomes with a treatment [68]. This information must be delivered in a way that is comprehensible to the patient.

#### Choosing a management strategy

Discussing the pros and cons of different management options will allow the patient to make an informed decision and select the approach that is best suited to him or her. To give the patient the greatest chance of success, it is critical that the initial advice is based on best-evidence recommendations and not biased toward a particular treatment strategy. This requires the clinician to synthesize the scientific evidence and their clinical experience to make specific recommendations for the individual patient. The rationale behind these recommendations should be as transparent as possible.

As the only randomized trial on the topic did not support a superior outcome with early ACLR [35], it is prudent to suggest a period of rehabilitation before surgical decision-making for most patients with ACL rupture. This strategy is also supported by the findings that preoperative rehabilitation improves postsurgical outcomes in those who go on to have an ACLR [62], [63], [64]. There is clinical agreement that patients who have functional instability after rehabilitation are likely to benefit from ACLR [69]. The rationale behind this is that frequent instability episodes can be prevented by an ACLR, thereby reducing potential damage to the menisci and cartilage.

Clinical practice patterns also show that many nonprofessional athletes choose to have an ACLR to enable return to pivoting sports [54], [69]. This does not reflect the current evidence [36], [41], [54]. Health-care professionals may favor one treatment over another, which could be reflected in the language used when they present management options to the patient. For example, consider the impact of a health-care professional using the following language: “you can have rehabilitation and change your lifestyle to avoid cutting and pivoting activities, or you can have surgery to fix your ACL and return to sport”. This phrasing is likely to leave the patient feeling as though ACLR is clearly the superior treatment. It would also obscure the important information that less than half of nonelite athletes return to competitive sport following ACLR, and that rehabilitation is usually more demanding following ACLR than with a nonsurgical approach.

Other factors such as concomitant injuries, access to treatment, patient preferences, and life plans may also influence the final decision. For example, external pressure will often give a professional athlete limited time for the decision-making process. It may therefore not be feasible to delay a surgical decision to see if they can return to sport with rehabilitation alone. On the other hand, if the athlete sustains an ACL rupture close to an important event and has no signs of functional instability, having an early ACLR would mean they could not compete in this event. He or she might therefore choose to participate in the event before considering whether surgery is likely to be of additional benefit to rehabilitation. The key to the treatment decision is to agree on a realistic treatment plan that sets the patient up with the best opportunity to achieve his or her goals.

### What is evidence-based ACL rehabilitation?

Evidence-based ACL rupture rehabilitation recommendations are presented in Table 3. Rehabilitation of patients with an ACL rupture should be led by a rehabilitation clinician who is experienced in rehabilitation of patients with ACL rupture. The physician should therefore refer the patient to a physical therapist or related health professional with this background. The number of visits needed will depend on the treatment plan and progress for each individual patient. Although it is not necessary for the rehabilitation clinician to monitor the execution of all exercises in a rehabilitation program, visits at least every two weeks may be required to ensure adequate progress. This requires that the patient has access to a location where he or she can perform the exercises in the rehabilitation plan, as the frequency of performing the exercises may range from daily to 2–3 times per week. Communication between the rehabilitation clinician and the physician is the key to optimal treatment, and the patient should be referred back to the physician if he or she (1) experiences symptoms of a new knee injury or repeated knee giving-way episodes, or (2) shows signs of complications that should be assessed. Examples of this include symptoms of a failed meniscal repair or failure to achieve full passive extension, which may be indicative of a cyclops lesion.

**Table 3:** Evidence-based ACL rupture rehabilitation recommendations.

Rehabilitation phase	Main goals	Description
**Preoperative phase** (for those who elect ACLR)	No knee joint effusion, full active and passive range-of-motion, 90% quadriceps strength symmetry	For patients who plan to undergo ACLR, preoperative rehabilitation should be performed to improve postsurgical outcomes [63], [64]. Rehabilitation should start as soon as possible after diagnosis. Preoperative rehabilitation follows the principles of acute and intermediate phase rehabilitation (described below), but deficits in passive knee extension range-of-motion and quadriceps strength should be specifically targeted as these factors are associated with poor postsurgical outcomes [70]. In patients with full range-of-motion, no effusion, and the ability to hop on one leg, preoperative rehabilitation with heavy resistance strength training and plyometric exercises is safe (3.9% adverse events) [71] and has benefits that extend at least two years after ACLR [62], [63].
**Acute phase** (after ACL rupture and/or ACLR)	No knee joint effusion, full active and passive range of motion, straight leg raise without lag	Treatments that target full passive extension and quadriceps muscle function should start the first day after ACL rupture or reconstruction. Active and passive range-of-motion exercises (e.g., quadriceps sets, active straight leg raise, prone hang, and heel slides), and effusion management by adjustment of loading are advocated in this phase [70]. Cryotherapy can be used to manage pain, but is not effective for the reduction of knee joint effusion [70]. As an additive to active exercises, high-intensity neuromuscular electrical stimulation (NMES) is effective in improving quadriceps strength after ACLR [72]. Postoperatively, evidence-based guidelines recommend both weight-bearing (closed kinetic chain) and nonweight-bearing (open kinetic chain) exercises [70].
**Intermediate phase** (after ACL rupture and/or ACLR)	Control of terminal knee extension in weight-bearing positions, 80% quadriceps strength symmetry, 80% hop test symmetry with adequate movement quality	This phase will integrate both neuromuscular training and muscle strength training [70]. Neuromuscular training aims to improve dynamic knee stability by establishing more beneficial proprioception and motor control strategies. Neuromuscular training is an umbrella term that includes perturbation training, balance training, agility drills, and plyometrics. A multi-modal approach is typically used and there is insufficient evidence to suggest that one type of training is superior to another. Blanchard and Glasgow [73] have described a theoretical model that can be used to progress neuromuscular exercises. An exercise starts with an internal focus. This means the focus is on achieving sound movement patterns in the exercise. Exercise characteristics, such as duration, speed, distance, or repetitions are then manipulated to increase the difficulty of the exercise. External factors, such as perturbations, hurdles or unstable surfaces are also added to progress the exercise. To enable skill transfer to sports, it is recommended to tailor the type of exercises to the patient by gradually introducing sport-specific skills [70]. The goal of a muscle strengthening program is to restore the muscle strength and power needed for participation in the patient's sport and desired recreational activities. Muscle strength exercises will start with an adjustment period that has lighter loads and a high number of repetitions and gradually progress to heavy loads with a lower number of repetitions. A strength training program that includes both bilateral and unilateral exercises, and that gradually progresses to principles for strength training for uninjured people, leads to better outcomes than training programs that consistently use a high number of repetitions [27]. The “+2 principle” can be used for load progression: once the patient can perform an extra two repetitions over the target number of repetitions, the load is increased in the next session [71].
**Late phase** (after ACL rupture and/or ACLR)	90% quadriceps strength symmetry, 90% hop test symmetry with adequate movement quality, maintain/build athletic confidence, progress sport-specific skills from closed skills with internal focus to open skills with external focus	Late phase rehabilitation should be individualized based on the patient's specific goals and athletic demands. The type of sport and physical activity that patients with an ACL rupture wish to participate in can vary widely; assessment of these athletic demands are therefore key to tailor a rehabilitation plan that leads to successful return to sport or activity. Typically, this phase includes impairment-specific heavy strength training, power and agility drills, and sport-specific exercises. After passing the criteria of a performance-based return to sport test battery, the athlete gradually resumes participation in unrestricted sports practice. This is achieved with a staged progression from modified training (e.g., noncontact only), to full training (unrestricted), to restricted participation in competition (by the number of minutes), to unrestricted participation in competition.
**Continued injury prevention phase** (after ACL rupture and/or ACLR)	Maintain muscle strength and dynamic knee stability, manage load	An injury prevention program should be performed at least twice per week as the patient gradually returns to sport and this should be maintained after a patient returns to full sport participation. Effective injury prevention programs exist for a variety of pivoting sports [23], and include lower limb strength exercises and training of low-risk movement patterns. Sports injury risk increases with sudden spikes in load (the combination of intensity and frequency of participation). Appropriate load-management may also be used to reduce the risk for knee re-injury and for injuries to other body parts [74].

Over the last decades, rehabilitation has moved from time-based protocols to individualized and criterion-based content and progression. There are five distinct phases of rehabilitation after ACL rupture (Table 3). Progress from one phase to the other only occurs when the patient meets specific clinical milestones. This criterion-based approach ensures that progression in rehabilitation does not surpass the functional and biological capacity of the knee. The move from time-based to criterion-based protocols also means that progress is not delayed unnecessarily.

### Factors that necessitate adjustments to the rehabilitation program

The principles of rehabilitation are similar for those who are treated with rehabilitation alone and those who elect to undergo ACLR. However, a shorter time-frame should be expected for those who do not have ACLR. After both ACL rupture and ACLR, concomitant injury and/or surgery may also require adjustments to the rehabilitation program. There are multiple combinations of concomitant injury presentations, and an injured structure may be nonoperatively managed or surgically treated using a variety of methods. For example, meniscus injuries may be surgically treated with a small resection, repair, or meniscus transplantation. These surgical methods require very different adaptations to the rehabilitation program [75]. Postsurgical restrictions will often be specified by the treating orthopedic surgeon, but clinicians who have less experience in managing these injuries postoperatively should consult a specialist if needed.

In patients, who undergo ACLR, the graft choice should be considered in postoperative rehabilitation. An ACLR can be performed with allograft (a donor graft) or autograft (usually from the patient's own ipsilateral patellar tendon or medial hamstring tendons). Rehabilitation after ACLR should address impairments from the ACL rupture *and* impairments from graft harvesting. ACLR with a patellar tendon autograft is associated with donor-site pain and quadriceps weakness [76], [77]. Rehabilitation clinicians should therefore monitor donor-site pain when the patient performs quadriceps strengthening exercises. After ACLR with hamstring grafts, the mechanical properties of the neo tendons may eventually recover with time [78]. To allow for healing, heavy resistance hamstring strengthening exercises are usually delayed for a period postoperatively [75], but there is no consensus on the optimal time frame for initiation of heavy resistance hamstring exercises. Hamstring peak muscle strength may also fully recover, but notable weakness in knee flexion strength at increased knee flexion angles is reported [79]. There is very little evidence to guide how rehabilitation should be tailored for patients who undergo less common methods of ACL surgery (e.g., ACLR with quadriceps tendon autograft, or ACL repair with internal bracing).

### How do I know when an individual is ready to return to sport after ACL rupture?

As outlined in the previous section, a gradual return to activity/sport is an integrated part of the rehabilitation progress. Different sports, and even different aspects within one sport, pose different demands to physical and psychological readiness. As the patient improves with rehabilitation, a gradual increase in sports participation is recommended to ensure the best transition back to full participation [80].

There are no high-level interventional studies that compare different criteria for return to sport. Current guidelines in this area are therefore informed by observational studies and expert opinion. Regardless of which activity/sport the patient is aiming to return to, there are three key considerations for the medical decision-making team: (1) is the athlete physically ready to participate in the activity/sport? (2) is the athlete mentally ready to perform in the activity/sport? (3) have we allowed enough time from injury/surgery for sufficient biological healing to occur?

#### Physical readiness

Assessment of the patient's knee function is a key aspect in the clinical decision of whether they should return to activity/sports. Pivoting sport athletes who pass specific criteria prior to return to sport have 4–6 times lower risk for re-injury [21], [22]. Grindem et al. [21] classified athletes as having passed or failed return to sport criteria with a return to sport test battery consisting of isokinetic quadriceps strength testing (≥90% of opposite leg), four single-legged hop tests (≥90% of opposite leg) and scores ≥90 on a scale from 0 (worst) to 100 (best) on a global rating scale of perceived function and the Knee Outcome Survey – Activities of Daily Living Scale. Athletes who failed any of the return to sport criteria were more likely to sustain a new knee injury (38%), compared to those who passed all return to sport criteria (6%). The test battery used by Kyritsis et al. [22] consisted of isokinetic quadriceps strength testing (≥90% of opposite leg), three single-legged hop tests (≥90% of opposite leg), and an agility test (≤11 s). Additionally, athletes had to complete on-field sport-specific rehabilitation. Among those who failed return to sport criteria, 33% sustained a graft rupture. In comparison, 10% of those who passed return to sport criteria ruptured their ACL graft.

Performance-based tests of muscle strength and single-legged hop ability have traditionally been the cornerstone of functional return to sport criteria [81]. Recently, there has been an increased focus on supplementing these tests with assessments of direction changes and reactive agility tests [80]. The rationale for these tests is to assess knee function during tasks that closely mimic those performed during sport participation. The choice of test will therefore depend on the sport to which the patient aims to return. Gradual introduction of sport-specific exercise in late phase rehabilitation can also be used to assess these aspects [80]. A gradual increase in training load is also a key component of the transition back to activity/sports [74]. Knee joint effusion and knee soreness rules are commonly used clinical markers to assess response to load [75], and can be used to guide progression throughout rehabilitation and return to sport. Once the athlete has resumed restricted sports activity, load can be monitored by multiplying session-rating of perceived exertion with session training minutes. A consistent progression in load is recommended to avoid exacerbation of symptoms and/or injury to the knee or other body parts [74].

#### Psychological readiness to return to sport

Psychological factors are highly associated with not returning to sport after ACLR. These factors can be related to the patient's competence, autonomy, and relatedness (e.g., low intrinsic motivation, low confidence, and high fear of re-injury) [82], [83]. Although psychological responses generally improve with rehabilitation, fear may increase, and remain a prominent emotion at the time of return to sport [82]. To assess psychological factors related to returning to sport, clinicians can use patient-reported tools, such as the anterior cruciate ligament return to sport after injury scale (ACL-RSI) [84]. Routine use of this tool can help the clinician and patient identifies important barriers to return to sport, and determine if additional treatment is required. This additional treatment may involve referral to a sports psychologist, but several strategies can be employed by the patient and non-psychologist clinician. There is currently very little research on interventions that effectively improve return to sport after ACL rupture through improved psychological readiness. Suggested strategies that target psychological factors include active goal-setting, having a role model the patient can relate to, relaxation techniques, and mental practice [83].

#### Biological healing

Over the last decades, clinical practice patterns have shifted toward earlier return to activity. Recent research has now highlighted that important biological healing processes are still ongoing at the time when athletes traditionally resume sports activities. Free tendon autografts are commonly used for ACLR. After systematically reviewing the literature, Claes et al. [85] observed that the time frame of the ligamentization process of these grafts was not well-defined, but may last more than 12 months after surgery. The process was prolonged in humans compared to animals, questioning previous knowledge on healing properties that was largely based on animal studies. After an isolated ACLR, cartilage quality and recovery in response to load is diminished [86]. Returning to sport before six months after surgery has been associated with poorer cartilage recovery after running, possibly implicating early exposure to high-impact activities in the development of post-traumatic knee osteoarthritis [86]. Early return to pivoting sports has also been associated with a high rate of knee re-injuries [21], [87]. It is unknown if the reduced risk of knee re-injury with more time after ACLR is explained by better biological healing or improved physical/psychological readiness. Up until 9 months after ACLR, a delay in return to sport by one month was associated with a 51% reduction in knee re-injury rates [21]. It is therefore recommended to delay return to pivoting sports until at least 9 months after ACLR.

## Summary

Following an ACL rupture the clinician and patient should, together, devise a treatment plan that addresses (1) rehabilitation, (2) appropriateness of ACLR, and (3) return to sport. Average long-term outcomes are similar following management of ACL rupture with rehabilitation alone or with ACLR and rehabilitation. Irrespective of treatment strategy, ACL rupture management should aim to restore knee function, address psychological barriers to sport/activity participation, reduce the risk of further injury and knee osteoarthritis, and optimize long-term QOL. Rehabilitation after an ACL rupture and/or ACLR should be individualized and criterion-based, with gradual return to sport/activity as an integrated part of rehabilitation progression. The three main factors in deciding readiness to participate in sport are (1) physical readiness, (2) psychological readiness, and (3) biological healing. Athletes who return to pivoting sport after ACLR can reduce their risk for re-injury by passing time-based and functional criteria before returning to sport.

**Practice points**

∗ The objective of ACL-injury management is to restore knee function, address psychological impacts, prevent further knee injury and osteoarthritis, and optimize long-term QOL;
∗ Current evidence suggests average long-term outcomes are similar following management of ACL rupture with ACLR and rehabilitation or with rehabilitation alone;
∗ Rehabilitation should start as early as possible after injury, and progress in rehabilitation is performed when the patient meets specific clinical or functional milestones;
∗ For some patients, not returning to sport can negatively impact long-term QOL, but returning to pivoting sport increases the risk of subsequent knee injury;
∗ Subsequent knee injury after ACL-injury is associated with poor long-term outcome, and reducing risk of subsequent knee injury should be a key priority when managing ACL-injured individuals;
∗ To reduce the risk for subsequent injury, pivoting sport athletes with ACLR should pass functional criteria for return to sport and delay full participation for at least nine months after surgery

**Research agenda**

∗ Research to determine optimal strategies for preventing subsequent knee injury and osteoarthritis after ACL rupture is required;
∗ Research is needed to establish return to sport guidelines for ACL-injured people who are treated with rehabilitation alone;
∗ Further research on late-phase rehabilitation and the efficacy of return to sport criteria is required;
∗ There is a need for research on interventions to improve psychological readiness to return to sport, and for research to evaluate the implications of such interventions on re-injury rates and long-term QOL;
∗ There is a need for further research to identify which patients will benefit most from management with or without ACLR

## Conflict of interest

None.
